# ZHX2 mediates proteasome inhibitor resistance via regulating nuclear translocation of NF‐κB in multiple myeloma

**DOI:** 10.1002/cam4.3347

**Published:** 2020-08-11

**Authors:** Jifeng Jiang, Yifeng Sun, Jiadai Xu, Tianhong Xu, Zhao Xu, Peng Liu

**Affiliations:** ^1^ Department of Hematology Zhongshan Hospital Fudan University Shanghai China

**Keywords:** multiple myeloma, NF‐κB, proteasome inhibitor resistance, ZHX2

## Abstract

**Background:**

Multiple myeloma (MM) is an incurable hematological malignancy. Although proteasome inhibitors and immunomodulators have significantly improved patient outcomes, some patients respond poorly to treatment and almost all patients will relapse. Mechanisms of proteasome inhibitor resistance in multiple myeloma have not been fully elucidated. ZHX2 is a transcription regulator degraded via proteasome and presents both oncogenic or tumor suppressive effect in different cancers, however, it is still unknown that the role of ZHX2 in myeloma. In this study, we aim to demonstrate the effect and mechanism of ZHX2 on proteasome inhibitor resistance in MM.

**Methods:**

GSE24080 gene expression profile datasets from Gene Expression Omnibus (GEO) were analyzed to evaluate the relationship between ZHX2 expression level and survival in MM. Expression of ZHX2 in human MM cell lines at baseline and after bortezomib (BTZ) treatment was determined by Western blotting (WB). The proliferation and apoptosis rate of MM cells treated with BTZ after the knockdown of ZHX2 were analyzed by flow cytometry. Nuclear translocation of NF‐κB after the knockdown of ZHX2 was evaluated by WB and immunofluorescence, and the expression of NF‐κB target genes was measured by real‐time quantitative PCR. Co‐immunoprecipitation (Co‐IP) and WB were used to detect the interaction of ZHX2 with NF‐κB.

**Results:**

We found that higher ZHX2 expression was correlated with poorer clinical outcomes of patients. In addition, ZHX2 expression was relatively higher in RPMI‐8226 and MM.1S cell lines and the level of ZHX2 protein was upregulated after BTZ treatment. Knockdown of ZHX2 significantly enhanced the sensitivity of MM cells to BTZ, inhibited nuclear translocation of NF‐κB, and reduced mRNA expression of NF‐κB target genes. It was also revealed that ZHX2 directly binds to NF‐κB.

**Conclusion:**

Our study showed that ZHX2 can promote proteasome inhibitor resistance in MM cells by regulating the nuclear translocation of NF‐κB.

## Graphical abstract

In this study, we aimed to demonstrate the effect and mechanism of ZHX2 on proteasome inhibitor resistance in MM. We found that ZHX2 can regulate the nuclear translocation of NF‐κB and is related to myeloma cell resistance to proteasome inhibitor.

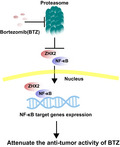

## 1 INTRODUCTION

Multiple myeloma is the second most common hematological malignancy, characterized by malignant plasma cell proliferation, infiltration, and secretion of a monoclonal immunoglobulin protein, which leads to end‐organ damage.^1^ As the most important component of first‐line treatment of multiple myeloma, proteasome inhibitors and immunomodulators have significantly improved patient outcomes.^2^ However, about a quarter of patients still have a poor response and almost all patients will relapse.^1^ Identifying novel mechanisms of resistance to these drugs is critical to further improving their efficacy.

The mechanisms of the antimyeloma effect of proteasome inhibitors include the following aspects. Proteasome inhibitors trigger endoplasmic reticulum stress and the unfolded protein response by accumulating misfolded and unfolded proteins, which induces apoptosis.^3^ Inhibition of NF‐κB signaling is also a critical effect of proteasome inhibitors.^4^ When the proteasome is inhibited, inhibitor of NF‐κB (IκB) cannot be degraded. This process leads to nuclear translocation of NF‐κB be inhibited and downstream signaling be inactivated.^5^ Mechanisms of resistance to proteasome inhibitors are related to the above process.^6^, ^7^ Most studies about proteasome inhibitors resistance focus on mutations and expression regulation in genes related to the ubiquitin‐proteasome system,^8^, ^9^ or molecules and pathways which can reduce proteotoxic stress.^10^, ^11^, ^12^ Some studies found that proteasome inhibitors can also activate antiapoptotic signaling, such as IGF‑1/IGF‑1R/AKT pathway, which results in drug resistance.^13^, ^14^ These studies suggest that the components of survival or antiapoptosis can be activated by proteasome inhibitors; however, some of other components remain to be found.

Recently, Zinc fingers and homeoboxes 2 (ZHX2) are reported as a von Hippel‐Lindau (VHL) E3 ubiquitin ligase protein substrate, and the degradation of ZHX2 is regulated by the ubiquitin‐proteasome system.^15^ ZHX2 can enhance the nuclear translocation of NF‐κB and promote tumorigenesis in renal cell carcinoma,^15^ however, studies also show that ZHX2 is a tumor suppressor in lung cancer and hepatocellular carcinoma,^16^, ^17^ therefore it is still unknown that the role of ZHX2 in myeloma.

As ZHX2 is a regulator of NF‐κB activation and can be degraded by proteasome, we hypothesize that ZHX2 may affect myeloma sensitivity to proteasome inhibitor. In this study, we reported that ZHX2 regulates the nuclear translocation of NF‐κB and proteasome inhibitor resistance in myeloma cell, providing a new mechanism of proteasome inhibitor resistance.

## 2 METHODS

### 2.1 Gene expression profiling and clinical data

Gene expression profiling and clinical data (GSE24080, Table S1) were obtained from Gene Expression Omnibus (GEO). The relationship between ZHX2 expression (probe 1557706_at) level and survival was analyzed by Kaplan‐Meier survival analysis. Patients received proteasome inhibitor‐based regimen and surviving more than 18 months were included in the analysis (n = 192).

### 2.2 Cell culture and transfection

Human MM cell lines RPMI 8226, MM.1S, U266, NCI‐H929 (Obtained from ATCC) used in this study were cultured in RPMI‐1640 medium (HyClone) supplemented with 10% fetal bovine serum (Gibco). Cells were cultured in a humidified atmosphere at 37°C with 5% CO_2_. Seed cells to be 2 × 10^5^ cells/mL at transfection. Lipofectamine™ 3000 Reagent (Invitrogen) and siRNA (Genomeditech) diluted in two tubes of Opti‐MEM medium and mixed well respectively. The diluted siRNA was mixed with the diluted Lipofectamine™ 3000 Reagent (1:1 ratio) and incubated for 15 minutes at room temperature. Cells were added to the mixture and cultured for 48 hour. SiRNA target sequences are shown in Table 1.

**TABLE 1:** SiRNA target sequences

Gene	sense (5ʹ‐3ʹ)	antisense (5ʹ‐3ʹ)
NC	UUCUCCGAACGUGUCACGUdTdT	ACGUGACACGUUCGGAGAAdTdT
ZHX2	CCGUAGCAAGGAAAGCAACAAtt	UUGUUGCUUUCCUUGCUACGGtt

### 2.3 Cell proliferation and apoptosis determined by flow cytometry

RPMI 8226 and MM.1S cell lines transfected with NC or ZHX2 siRNA were seeded in 6‐well plates and treated with BTZ for 48 hours, then cells were collected. For the analysis of proliferation, cells were incubated with 5‐ethynyl‐2′‐deoxyuridine (EDU, RiboBio) for 2 hour and fixed in 4% paraformaldehyde (Servicebio) for 15 minutes. After washed in PBS (HyClone), Triton X‐100 (Beyotime) was added to the cell suspension for 15 minutes. Finally, the cells were incubated with 200 μL of staining solution following the manufacturer's instructions. For the analysis of apoptosis, treated cells were stained with 5 μL of FITC‐conjugated Annexin V and propidium iodide (PI) for 15 minutes. The samples were analyzed by flow cytometry.

### 2.4 Immunofluorescence (IF) assay

Cells were seeded and transfected with NC or ZHX2 siRNA in 6‐well plates. After 48 hour, cells were collected and fixed with 4% paraformaldehyde, then permeabilized with Triton X‐100, and blocked with blocking buffer (Beyotime). Samples were incubated with the primary antibody anti‐P65 (NF‐κB, CST) and fluorescein‐labeled secondary antibody (CST), and then DAPI (Beyotime) was used to stain the nucleus.

### 2.5 Quantitative RT‐PCR

Total RNA was extracted with TRIzol (Invitrogen) from the cell samples. RNA samples were reverse transcripted by RevertAid RT Reverse Transcription Kit (Thermo fisher scientific) according to the manufacturer's instructions. For quantitative mRNA analysis, TB Green^®^ Premix Ex Taq™ II (Takara) was used to determine the expression levels of ZHX2 and NF‐κB target genes. The relative expression level of mRNA was calculated using the 2^‐ΔΔCt^ method. Primer sequences are shown in Table 2.

**TABLE 2:** Primer sequences for qRT–PCR

Gene	Forward (5ʹ‐3ʹ)	Reverse (5ʹ‐3ʹ)
GAPDH	GAAGGTGAAGGTCGGAGTC	GAAGATGGTGATGGGATTTC
ZHX2	GATCAGATAGCTGGAGTCAGGC	CACAGCAGTTCTAACAGACTTCC
IL‐6	AGACAGCCACTCACCTCTTCAG	TTCTGCCAGTGCCTCTTTGCTG
IL‐6R	CCCCTCAGCAATGTTGTTTGT	CTCCGGGACTGCTAACTGG
BCL‐2	ATCGCCCTGTGGATGACTGAGT	GCCAGGAGAAATCAAACAGAGGC
VEGFA	CTGCTGTCTTGGGTGCATTGG	TCACCGCCTCGGCTTGTC
PDGF	GTGAACGCAGTGCAGACTGT	AGGTGTAGGTCCCCGAGTCT
FGF	GAAGTTCAAATGCCCTTCCA	CCAGCTGGTATGTGTGGTTG
ICAM1	AGCGGCTGACGTGTGCAGTAAT	TCTGAGACCTCTGGCTTCGTCA

### 2.6 Western blot and Co‐immunoprecipitation (Co‐IP)

Cell lysate was prepared using RIPA (Beyotime) or Pierce IP lysis buffer (Thermo fisher scientific) containing protease inhibitor cocktail. Protein quantification was performed by BCA assay (Beyotime). Membrane was blocked in 5% skin‐milk for 1 hour and then incubated with primary antibody overnight at 4C. Horseradish peroxidase‐conjugated secondary antibody (Beyotime) was then added for 1 hour at room temperature. Briefly, protein supernatant for Co‐IP was incubated with Protein A/G PLUS‐Agarose (Santa Cruz Biotechnology) beads for 3 hour at 4°C. The supernatant was transferred to a new tube, added anti‐ZHX2 or anti‐IgG antibodies, and incubated for 1 hour at 4°C. The mixture was incubated with beads under rotary agitation overnight at 4°C, then centrifuged and discarded the supernatant. Beads were collected and washed for three times. The 2 × loading‐buffer was added to beads and the mixture was heated at 100℃ for 10 minutes. Protein‐antibody complexes were determined by immunoblot. The following primary antibodies were used: ZHX2(GeneTex), NF‐κB (CST), Tubulin (Beyotime), and IgG (ABclonal).

### 2.7 Statistical analysis

All data were presented as mean ± standard deviation from three independent experiments and the significance wad analyzed using Student's *t*‐test. Survival analysis was evaluated using Kaplan–Meier plots and Log‐Rank test. *P*‐values <.05 were considered statistically significant. For statistical analyses, we used SPSS 22.0 software.

## 3 RESULTS

### 3.1 Higher ZHX2 expression is correlated with poorer clinical outcomes of patients

**FIGURE 1:**
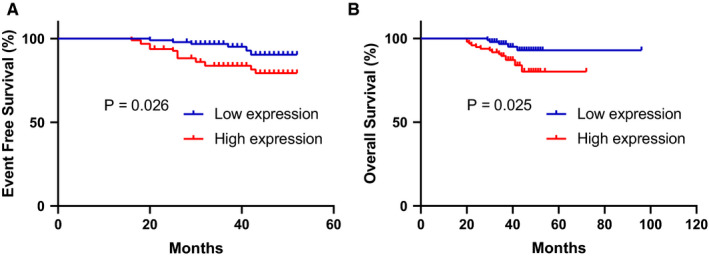
High ZHX2 expression is correlated with poorer clinical outcomes of patients. Kaplan‐Meier survival curve for ZHX2 expression in the GSE24080 (probe 1557706_at) cohort of newly diagnosed MM patients (n = 192) receiving BTZ. (A)Overall survival (OS). (B) Event‐free survival (EFS)

We analyzed the data of expression profiling array (GSE24084; probe 1557706_at) in GEO datasets. We found that in bortezomib‐treated patients who survived more than 18 months with higher ZHX2 expression showed a shorter event‐free survival (*P* = .025) and overall survival (*P* = .026) (Figure 1A and B).

### 3.2 ZHX2 expression is relatively higher in RPMI‐8226 and MM.1S cell lines and protein levels of ZHX2 can be upregulated by bortezomib

**FIGURE 2:**
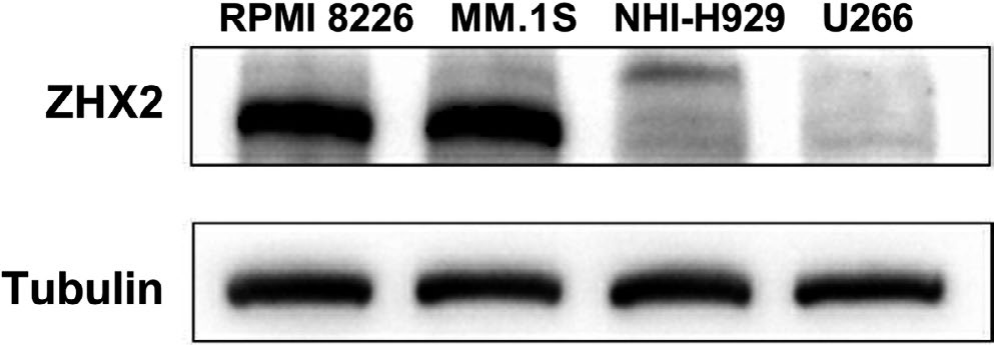
Expression of ZHX2 in different MM cell lines. Expression of ZHX2 in MM cell lines was detected by Western blot

**FIGURE 3:**
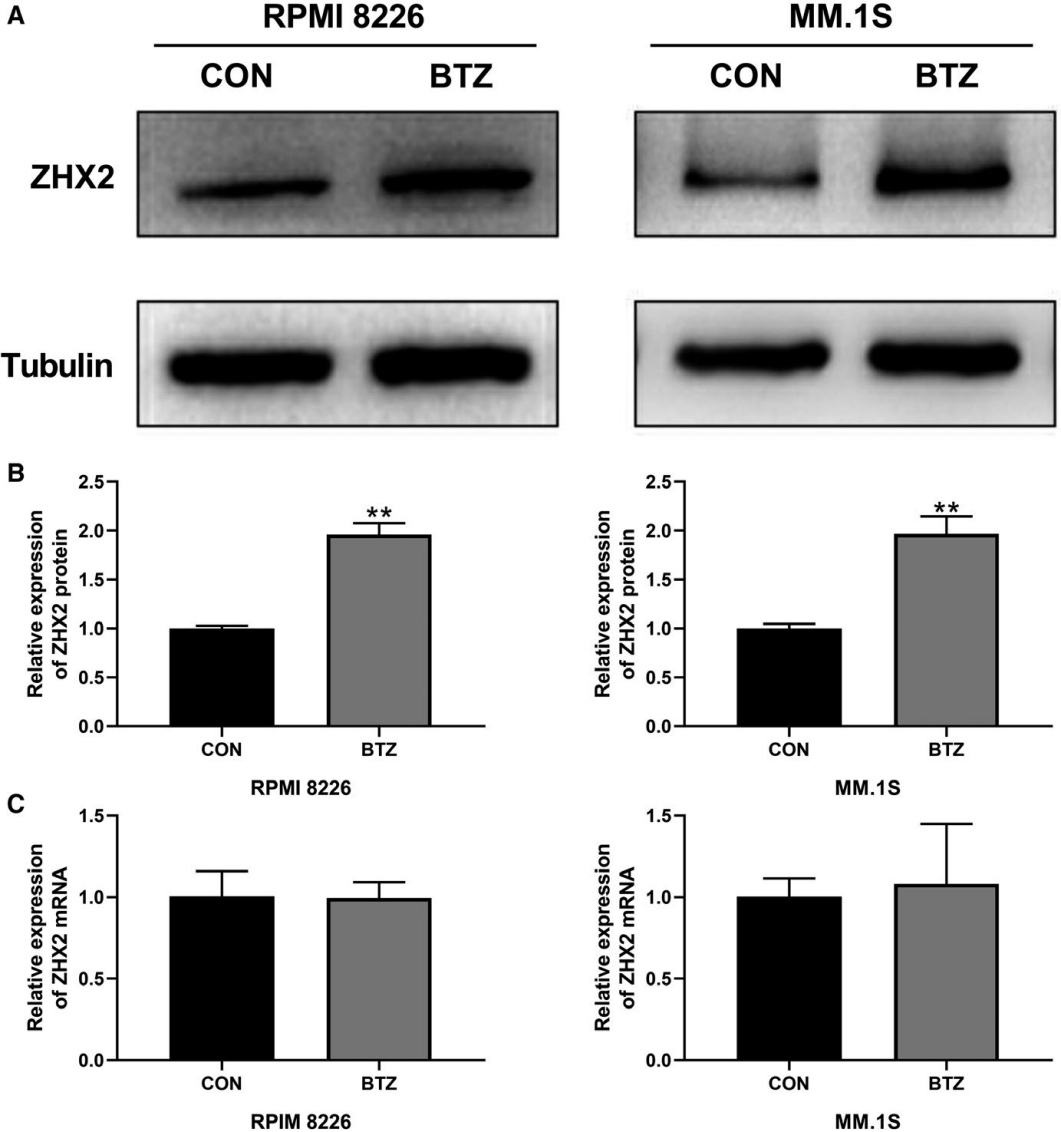
ZHX2 expression was upregulated by BTZ. (A) RPMI‐8226 and MM.1S cells were treated with 5 nmol/L BTZ for 48 hour and ZHX2 was detected by Western blot. (B) Quantification of ZHX2 protein levels. (C) Quantification of ZHX2 mRNA levels. Data were expressed as the mean ± SD (n = 3). *P < .05, **P < .01, compared to the NC group

We detected the expression of ZHX2 in four myeloma cell lines, RPMI‐8226, MM.1S, NCI‐H929, and U266. ZHX2 expression was relatively higher in RPMI‐8226 and MM.1S, while U266 did not express ZHX2 (Figure 2). As shown in Figure 3A and B, when RPMI‐8226 and MM.1S cell lines were treated with bortezomib (5 nmol/L) for 48 hour, the level of ZHX2 protein was significantly upregulated, but mRNA levels of ZHX2 were unchanged (Figure 3C).

### 3.3 Knock‐down ZHX2 enhanced the sensitivity of MM cell to BTZ

**FIGURE 4:**
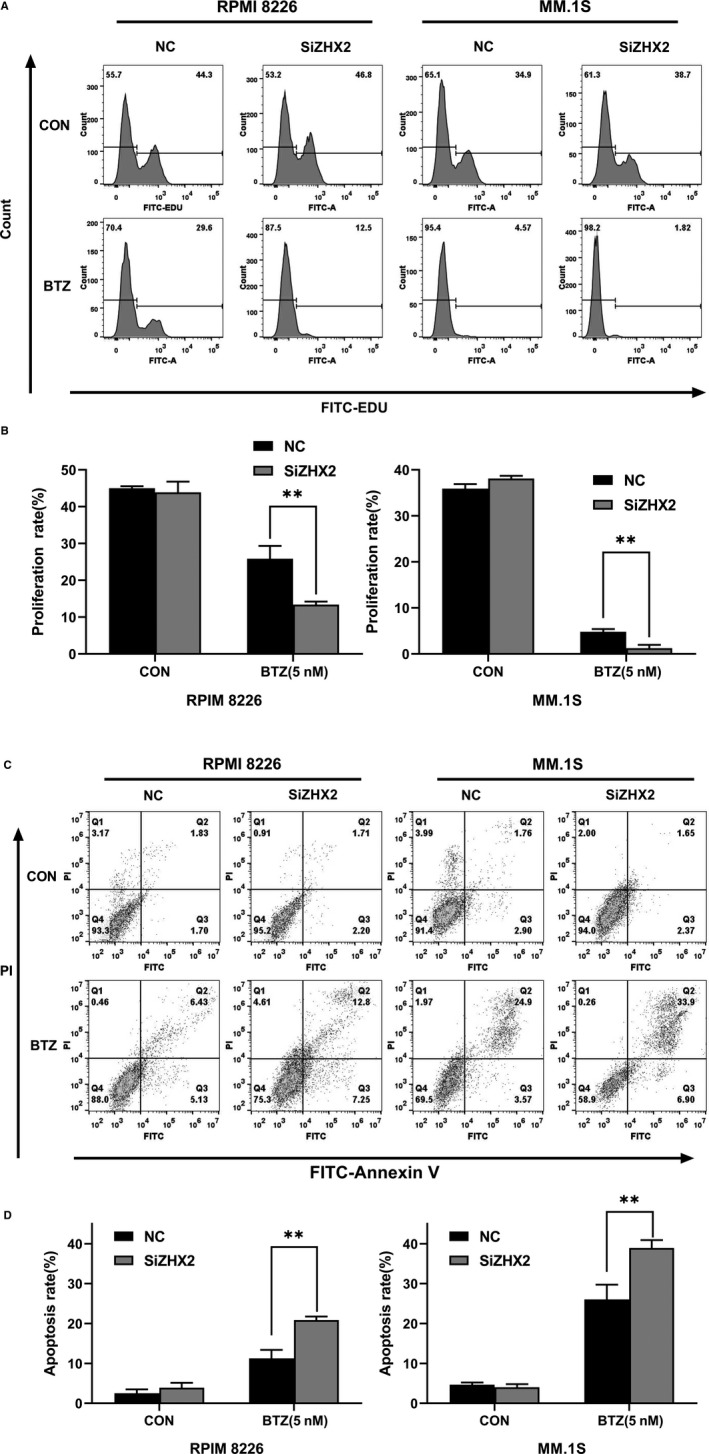
Knock‐down ZHX2 enhanced the sensitivity of MM cell to BTZ. (A) Cells were incubated with EDU and analyzed by flow cytometry. (B) The percentage of cell proliferation. (C) Flow cytometry after staining with PI and Annexin V to determine cell apoptosis. (D) The percentage of cell apoptosis. Data were expressed as the mean ± SD (n = 3). *P < .05, **P < .01, compared to the NC group

After transfected with NC or ZHX2 siRNA (The efficiency of knockdown is shown in Figure S1), RPMI‐8226 and MM.1S cells were treated with BTZ for 48 hour, and the results showed that the proliferation rate of the si‐ZHX2 group was lowered compared with the NC group (Figure 4A and B). Moreover, the apoptosis induced by BTZ was also enhanced in this group (Figure 4C and D). These findings indicated that ZHX2 decreased the sensitivity of myeloma to BTZ.

### 3.4 ZHX2 regulated nuclear translocation of NF‐κB

**FIGURE 5:**
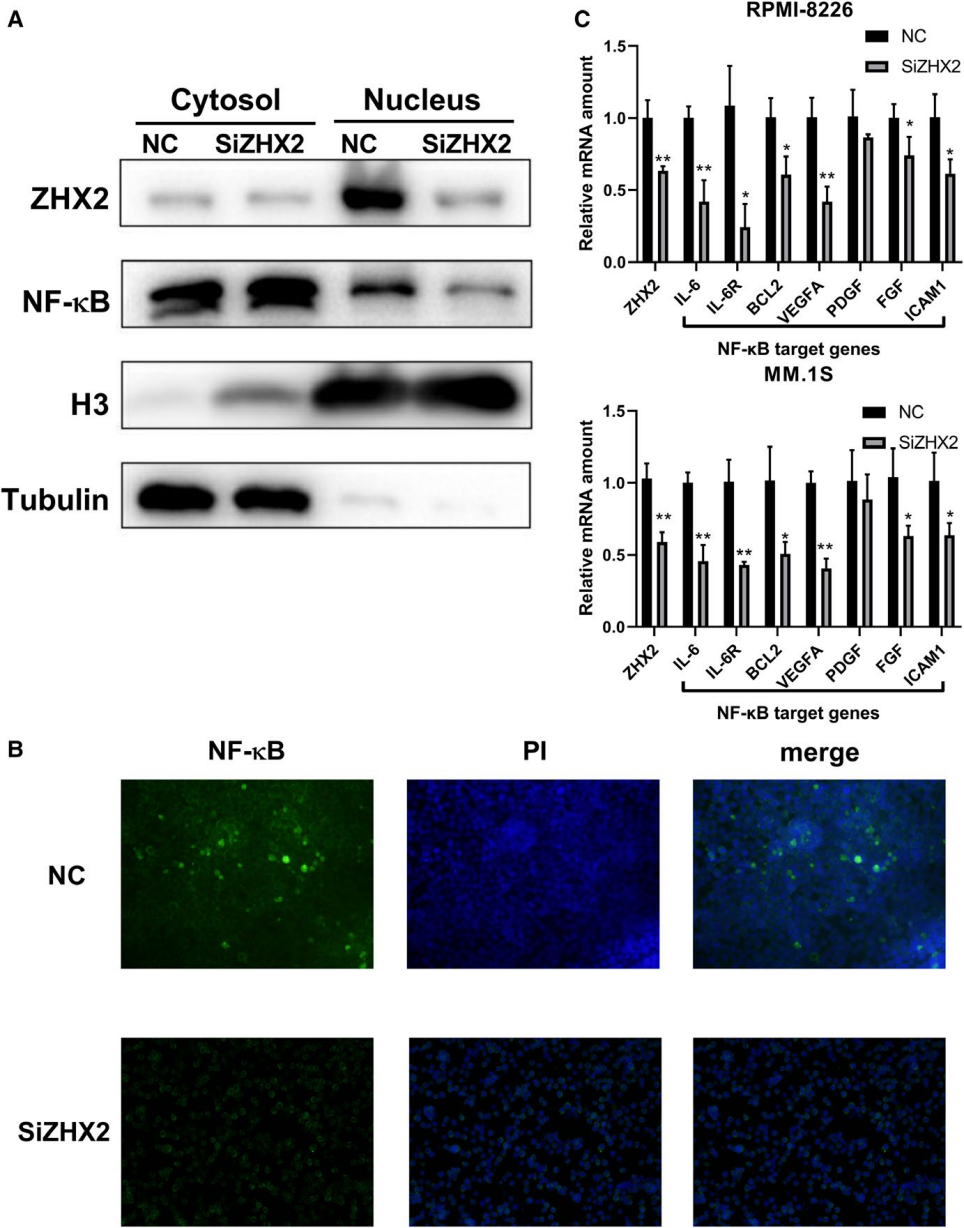
ZHX2 regulated nuclear translocation of NF‐κB and its target genes expression in MM cells. (A and B) Nuclear translocation of NF‐κB in cytosol and nucleus after transfected with ZHX2 siRNA for 48 hour. (A) Western blot of nuclear translocation of NF‐κB. (B) Immunofluorescence analysis for nuclear translocation of NF‐κB. (C) The mRNA levels of NF‐κB target genes after transfected with ZHX2 siRNA for 48 h were analyzed by RT‐qPCR. Data were expressed as the mean ± SD (n = 3). *P < .05, **P < .01, compared to the NC group

To clarify the mechanisms of ZHX2 affecting the sensitivity of myeloma cell to BTZ, we determined NF‐κB expression in cytoplasm and nucleus after ZHX2 knock‐down. We found that the amount of NF‐κB in the nucleus was significantly decreased in the si‐ZHX2 group and there was no change in the amount of NF‐κB in the cytoplasm. Therefore, these results suggested that ZHX2 regulated the nuclear translocation of NF‐κB (Figure 5A and B).

### 3.5 ZHX2 regulated the expression of NF‐κB target genes

To explore whether ZHX2 regulated the transcription of NF‐κB target genes, we detected the expression of NF‐κB target genes in RPMI‐8226 and MM.1S cell lines transfected with ZHX2 siRNA or NC siRNA. We observed that the expression of IL‐6, IL‐6R, BCL‐2, VEGFA, PDGF, and FGF was decreased (*P* *<* .05) in the si‐ZHX2 group (Figure 5C).

### 3.6 ZHX2 directly interacted with NF‐κB

**FIGURE 6:**
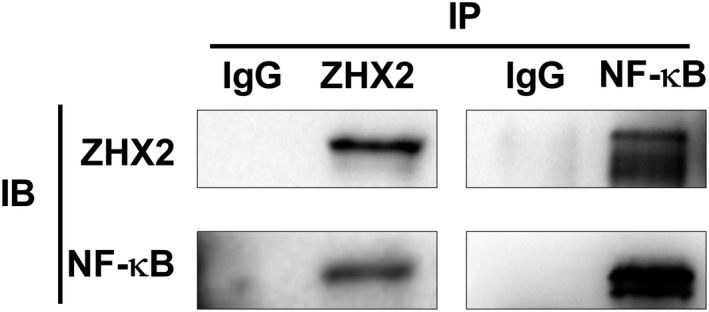
ZHX2 interacted with NF‐κB. Co‐IP for the interaction of ZHX2 with NF‐κB in RPMI‐8226 cells

We then tested whether ZHX2 directly interacted with NF‐κB. We performed Co‐IP for the ZHX2 and NF‐κB in the RPMI‐8226 cell. The results showed that ZHX2 could physically bind to NF‐κB (Figure 6).

## 4 DISCUSSION

Although the outcome of MM has been improved by proteasome inhibitors and immunomodulators, MM is still considered an incurable hematological malignancy.^18^ It is important to find the biomarker or target of drug resistance in MM. We examined the data of expression profiling array in GEO datasets. We observed that higher ZHX2 expression was associated with poorer outcomes in MM patients who received bortezomib‐based treatment regimen. This result suggested that ZHX2 was related to proteasome inhibitors resistance.

As a representative drug of proteasome inhibitors, BTZ inhibits the 20S proteasome and was approved for the treatment of myeloma and mantle cell lymphoma by FDA.^19^ However, solid tumors is poorly response to proteasome inhibitors, and this may be explained by the special antitumor mechanism of proteasome inhibitors.^19^ Myeloma cells usually synthetize large amounts of immunoglobulins, therefore they rely heavily on the proteasome system to maintain cellular homeostasis and are sensitive to proteasome inhibitors.^7^ NF‐κB is also a target of proteasome inhibitors, which provides survival and proliferation signals in myeloma cells.^4^, ^6^ Xue et al^20^ reported that bortezomib inhibited the NF‐κB pathway and induced apoptosis in prostate cancer cell lines; however, prostate cell resistance to BTZ developed after repeated treatment. Interestingly, it was found that proteasome inhibitors can upregulate oncogene in lung and esophageal cancer cell lines.^20^, ^21^ Evidence also shows that the degradation of oncoproteins can be decreased in cancer with the deficiency of the ubiquitin ligase component. Such as the degradation of HIF‐1α and ZHX2 can be inhibited in VHL deficiency renal carcinoma.^15^, ^22^ These results suggest that the inhibition of proteasome may lead to the upregulation of oncoproteins and we believe that this process may attenuate the antitumor effect of proteasome inhibitors. Our results are consistent with the hypothesis, as the Figure 3 showed that protein levels of ZHX2 in myeloma cell lines treated with bortezomib was higher than the control group.

ZHX2 was first reported by Kawata et al,^23^ as a transcriptional repressor, which consists of 837 amino acid residues, including two zinc finger motifs and five homology domains, and can interact with the transcription factor NF‐YA to regulate gene expression. Later studies show that ZHX2 is a tumor suppressor in hepatocellular carcinoma and lung cancer.^17^, ^24^ However, ZHX2 can play an oncogenic role in kidney cancer.^15^ It is also found that ZHX2 promotes cell survival and reduces apoptosis in non‐tumor cells, such as macrophage.^25^ These findings suggest that the effect of ZHX2 varies among different tumors. We found that knockdown ZHX2 enhanced the sensitivity of MM cell to bortezomib. The proliferation rate was lower and the apoptosis rate was higher in the si‐ZHX2 group treated with bortezomib than the NC group.

NF‐κB can be continuously activated in a variety of malignant tumors to promote cell proliferation, inhibit apoptosis, enhance cell migration and invasion, and induce angiogenesis and metastasis.^26^ There are two types of NF­κB pathways, canonical and non‐canonical. Canonical NF­κB pathway mainly consists of p50 and p65 which are activated by inflammatory cytokines, pathogen‐associated molecules, and antigen receptors.^26^ The activation of NF‑κB signaling requires the ubiquitin‐proteasome system to degrade the inhibitor of NF‑κB (IκB), and this process can be as a target of proteasome inhibitors to inhibit NF‑κB activation.^5^, ^27^ Consistent with the report of Yao et al,^22^ we also found that ZHX2 directly interacted with canonical NF‐κB component (p65) and knock‐down ZHX2 decreased the nuclear translocation of NF‐κB in MM cells. We speculated that bortezomib inhibited IκB degradation and NF‐κB activation while decreased the degradation of ZHX2, which attenuated the inhibitory effect of bortezomib on NF‐κB activation. This may partly explain the mechanism of ZHX2 regulated sensitivity to proteasome inhibitor in MM cells.

NF‐κB pathway regulates a large number of genes expression and is related to pathogenesis and progression of MM.^4^ IL‐6/IL‐6R, as the target genes of NF‐κB, has been well studied for its effect on proliferation and survival in MM.^28^ Bcl‐2 is one of the pro‐survival proteins in the Bcl‐2 protein family and is overexpressed in myeloma cell lines and patients samples.^29^, ^30^ Bcl‐2 can be considered as a target for the treatment of MM.^31^ We observed that IL‐6/IL‐6R, Bcl‐2, and other NF‐κB target genes were downregulated after knockdown ZHX2, indicating the role of ZHX2 on regulating the transcriptional activity of NF‐κB and biological functions in MM.

**FIGURE 7:**
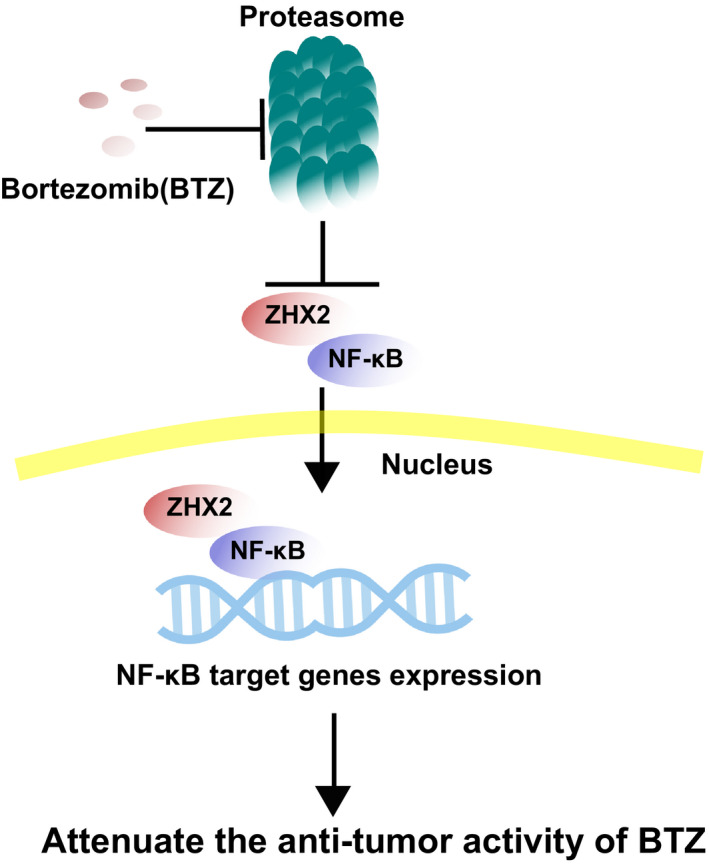
The mechanism of ZHX2 promoting proteasome inhibitor resistance. When bortezomib inhibits the proteasome, the degradation of ZHX2 is decreased. Accumulated ZHX2 binding to NF‐κB enhancing nuclear translocation of NF‐κB and activation of NF‐κB target genes expression, which can attenuate the antitumor activity of proteasome inhibitor

In conclusion, our study showed that ZHX2 can regulate the nuclear translocation of NF‐κB and is related to myeloma cells’ resistance to proteasome inhibitor. Our work illustrated a new mechanism of proteasome inhibitors resistance in MM (Figure 7). We will look for interventions that can overcome ZHX2‐mediated resistance to proteasome inhibitors in further studies.

## CONFLICT OF INTEREST

The authors declare that they have no conflicts of interest.

## AUTHORS CONTRIBUTIONS

Peng Liu contributed to the conception and design of the study and revised the manuscript; Jifeng Jiang conducted the experiments and wrote the manuscript, Yifeng Sun also performed the experiments; Jiadai Xu, Tianhong Xu, and Zhao Xu analyzed the data.

## Supporting information

Fig S1

Table S1

## Data Availability

The data and materials used are available when requested.
