# Supplementary figures and images for: ZHX2 mediates proteasome inhibitor resistance via regulating nuclear translocation of NF‐κB in multiple myeloma
Source: Cancer Med. 2020 Aug 11;9(19):7244–52. doi: 10.1002/cam4.3347 (PMC7541163; doi:10.1002/cam4.3347)

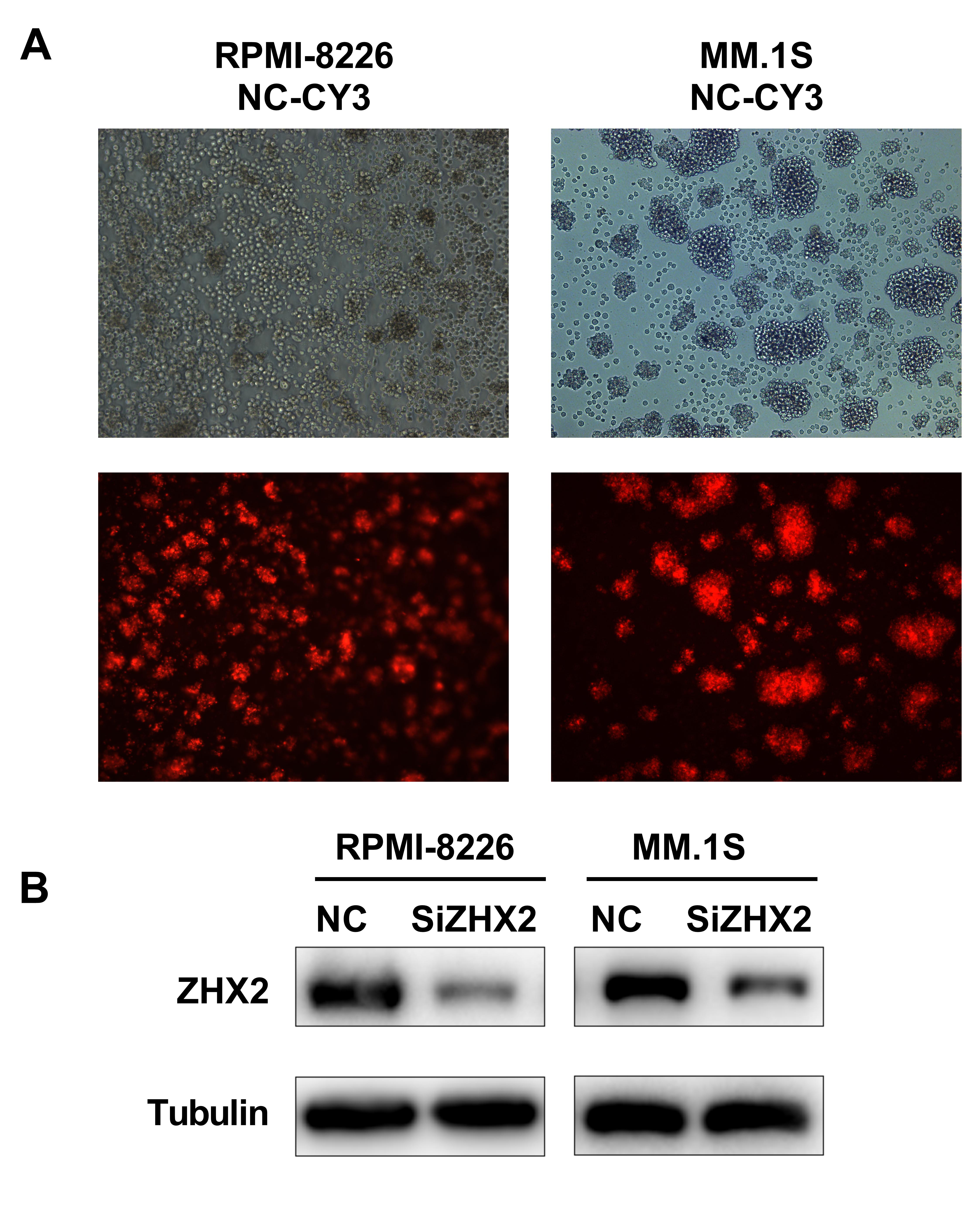

Supplement: Supplementary file 1 — Fig S1 [file CAM4-9-7244-s001.jpg]
